# Association study of monoamine oxidase A/B genes and schizophrenia in Han Chinese

**DOI:** 10.1186/1744-9081-7-42

**Published:** 2011-10-06

**Authors:** Yi-Liang Wei, Cai-Xia Li, Sheng-Bin Li, Yao Liu, Lan Hu

**Affiliations:** 1 Department of Forensic Science, School of Medicine, Xi'an Jiaotong University/Key Laboratory of Ministry of Public Health for Forensic Science, Xi'an 710061, PR China; 2 Key Laboratory of Environment and Genes Related to Diseases, Xi'an Jiaotong University, Ministry of Education, Xi'an 710061, PR China; 3 Institute of Forensic Science, Ministry of Public Security, Beijing 100038, PR China; 4 Key Laboratory of Forensic Genetics, Ministry of Public Security, Beijing 100038, PR China

## Abstract

**Background:**

Monoamine oxidases (MAOs) catalyze the metabolism of dopaminergic neurotransmitters. Polymorphisms of isoforms MAOA and MAOB have been implicated in the etiology of mental disorders such as schizophrenia. Association studies detected these polymorphisms in several populations, however the data have not been conclusive to date. Here, we investigated the association of *MAOA *and *MAOB *polymorphisms with schizophrenia in a Han Chinese population.

**Methods:**

Two functional single nucleotide polymorphisms (SNPs), rs6323 of *MAOA *and rs1799836 of *MAOB*, were selected for association analysis in 537 unrelated schizophrenia patients and 536 healthy controls. Single-locus and Haplotype associations were calculated.

**Results:**

No differences were found in the allelic distribution of rs6323. The G allele of rs1799836 was identified as a risk factor in the development of schizophrenia (*P *= 0.00001). The risk haplotype rs6323T-rs1799836G was associated with schizophrenia in female patients (*P *= 0.0002), but the frequency difference was not significant among male groups.

**Conclusions:**

Our results suggest that *MAOB *is a susceptibility gene for schizophrenia. In contrast, no significant associations were observed for the *MAOA *functional polymorphism with schizophrenia in Han Chinese. These data support further investigation of the role of MAO genes in schizophrenia.

## Background

Schizophrenia is a chronic mental disorder characterized by abnormalities in the perception or expression of reality. Onset of symptoms typically occurs in young adulthood. Global estimates for lifetime prevalence of schizophrenia are 4.0‰-7.0‰ [1,2]. The disease has been ascertained with a high level of heritability by twins and adoption studies [3]. Many candidate genes have been found, such as *DAOA *(G72), *DTNBP1 *(dysbindin), *COMT*, with each having small effects in genome-wide association studies. Many of these genes have also been implicated in the etiology of bipolar disorder, as both diseases have some manifestations in common [4,5]. Schizophrenic patients display increased dopamine activity in the mesolimbic pathway of the brain, and often present with additional conditions such as major depression and anxiety disorders [6].

Monoamine oxidase (MAO), a mitochondrial enzyme, plays a vital role in the inactivation of neurotransmitters. MAOA and MAOB are two biochemically distinct forms of this enzyme that are encoded by distinct genes located adjacently on the X chromosome in opposite direction [7]. While MAOA and MAOB are different in metabolic substrates and inhibitor specificities, they equally contribute to the deamination of dopamine. According to the dopamine theory on the pathogenesis of schizophrenia, low activity of MAO is a risk factor in the development of the disorder [8,9].

Polymorphisms of the *MAOA *gene have been investigated in several psychiatric illnesses including schizophrenia [10], major depressive disorder (MDD) [11] and bipolar affective disorder (BPD) [12]. Examples of *MAOA *polymorphisms include rs6323, rs1800466, rs1799835, and rs1465108. The functional polymorphism (rs6323), located in exon 8, is associated with altered enzyme activity and has been extensively investigated in association studies. Synonymous substitution of T to G at this location promotes MAOA activity [13,14]. MDD patients with genotype G or G/G at this site have a significantly lower magnitude of placebo response than those with T, G/T or T/T [15]. A recent study implied that the T allele was associated with schizophrenia in Chinese males [16], however this association was not confirmed in a meta-study comprising Caucasian, Japanese, and Han Chinese [17].

*MAOB*, located adjacent to *MAOA *on the opposite strand at chromosome Xp11.23, is involved in the breakdown of dopamine in the brain. A non-coding single nucleotide polymorphism (SNP) (rs1799836) in intron 13 is associated with Parkinson's disease [18], and is also significantly associated with reduced negative emotionality [19]. This A/G (A644G) substitution is responsible for altered enzyme activity with tissue specificity [20-23]. Further, a case-control study by Gassó et al. indicates that the G allele is a risk factor for developing schizophrenia in a Spanish population [9].

**Figure 1:**
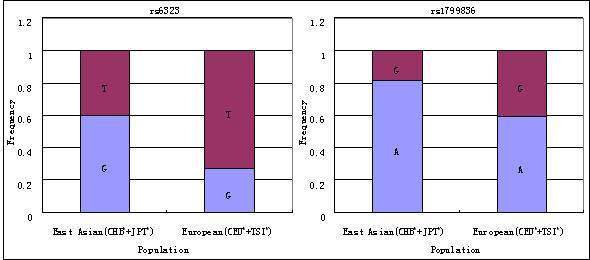
**Population distribution of rs6323 and rs1799836^a^**. ^a^Data cited from Hapmap Database. ^b^CHB: Han Chinese in Beijing, China; JPT: Japanese in Tokyo, Japan; CEU: Utah residents with Northern and Western European ancestry from the CEPH collection; TSI: Tuscan in Italy.

Genetic polymorphisms associated with altered enzyme activity may play a significant role in the etiology of schizophrenia. Here, we investigated the association of two representative functional polymorphisms, rs6323 of *MAOA *and rs1799836 of *MAOB*, with the development of schizophrenia in Han Chinese. The full list of genes and selected polymorphisms is outlined in Table 1. These specific polymorphisms have been investigated in previous studies, however the results have not been conclusive. Here, we investigate associations of these polymorphisms utilizing a large sample size allowing generation of significant data. Additionally, as emerging reports indicate that *MAOA *and *MAOB *may have gender-specific roles in the development of several psychiatric disorders [9,12,17,19,24], we have included differences in gender in our statistical analysis. The MAO genes are located on the X chromosome, therefore males are hemizygotes and haplotypes formed by rs6323 and rs1799836 can be explicitly assigned in male participants. Finally, differences in allele frequency distributions of rs6323 and rs1799836 have been identified across multiple populations [25]. In general, such allele frequency differences can lead to association errors (type I or II) if there is population stratification in the samples. The same allele may confer a different risk for disease in one population as compared to another due to variations in genetic background or linkage disequilibrium (LD) patterns. As significant genetic difference between European and East Asian populations exist (Figure 1; δ_rs6323 _= 0.225, δ_rs1799836 _= 0.330), we have limited our investigation to Han Chinese. Accounting for these parameters, a case-control study was proposed to investigate the associations of rs6323 and rs1799836 polymorphisms with schizophrenia, and to generate a comparison of the relative contribution of each gene. Genetic association was examined simultaneously on alleles, genotypes and haplotypes.

**Table 1:** Details of SNPs


**dbSNP**	**Position^a^**	**Gene symbol**

rs6323	chrX: 43591036	*MAOA*
rs1799836	chrX: 43627999	*MAOB*

^a^Map to Genome Build 37.1 in dbSNP.

## Material and methods

### Sample collection

Han Chinese subjects (n = 537 patients diagnosed with schizophrenia by a group of experienced psychiatrists using DSM-IV criteria, including 294 men and 243 women), were recruited from Xi'an Mental Health Centre. Matched controls (n = 536, including 284 males and 252 females) based on geographic origin, gender ratio and age and without any family or personal history of neurological disorders, were recruited from hospital staff, students, and the general population. The patient group had an age distribution of 12 and 72 years (mean ± SD: 29.95 ± 8.69 years), while the control population had an age distribution of 13 and 70 years (mean ± SD: 30.34 ± 11.63 years). A questionnaire was administered to obtain demographic information from all of the subjects, including sex, age, ethnicity, personal medical history, and family history of neurological disease. Clinical data of all patients was obtained from hospital check-in records.

Whole blood samples were obtained from all patients, and buccal swabs and blood spots were collected from healthy controls. This study was approved by the Xi'an Jiaotong University Ethics Committee. Written informed consent was obtained from all participants. Another previous study using part of these samples has been published [26].

### Experimental procedures

DNA was isolated from 1 ml of blood using the QIAamp^® ^DNA Blood Midi Kit and from saliva or blood spot samples by MagAttract^® ^DNA Mini M48 Kit. Genotypes of rs6323 were determined by sequencing. The forward primer 5' -GACCTTGACTGCCAAGAT-3' and reverse primer 5' -CTTCTTCTTCCAGAAGGCC-3' were used to amplify a 130 bp fragment. Genotype analysis of locus rs1799836 was performed by adopting the method of A/G-specific oligonucleotide polymerase chain reaction, which was described previously [9]. A- and G- specific oligonucleotides 5'-CACTGGCAAATAGCAAAAGT-3' and 5'-CACTGGCAAATAGCAAAAGC-3' were used as reverse primers and 5'-GGATTTACTTTGCAGGCACC-3' was used as a forward primer, the products' size was 663 bp. For quality control of this method, nearly 8% of samples (80 samples) were randomly selected to take re-sequencing.

### Statistical analysis

Data were analyzed using the SPSS v15.0 software package (SPSS Inc., Chicago IL). Mean and standard deviations were computed for continuous variables. For male data, allelic frequency distributions between patients and matched controls were analyzed using the χ^2 ^test and Fisher's exact test. Hardy-Weinberg Equilibrium was examined by χ^2 ^test. Calculations of odds ratio and confidence limits for the risk alleles and haplotypes were implemented in the SPSS program. Haploview was used to analyze Hardy-Weinberg Equilibrium, linkage disequilibrium and disease association in females [27]. Test power was estimated using G*Power 3.0 [28] at the significant level of 0.05 (α = 0.05) and the effect size of 0.3 for all statistical tests, and Bonferroni correction (α') was applied for multiple tests in comparison analysis.

## Results

### Single-locus frequency distribution and association analysis

Frequency of SNPs rs6323 and rs1799836 were analyzed in case and control groups. Allelic frequency distributions of markers are presented in Table 2. Each distribution was in accordance with Hardy-Weinberg Equilibrium, respectively, for males and females in both case and control groups (MAF > 0.1, *P *> 0.05). Results obtained from A/G-specific oligonucleotide polymerase chain reaction were in complete concordance with the re-sequencing results.

**Table 2:** Allelic frequencies of rs6323 and rs1799836 in schizophrenia patients and control subjects


**Allele**	**Schizophrenia (n, %)**			**Control (n, %)**		
	**Male**	**Female**	**Total**	**Male**	**Female**	**Total**

rs6323						
G	167/294 (56.8)	262/486 (53.9)	429/780 (55.0)	160/284 (56.3)	276/504 (54.8)	436/788 (55.3)
T	127/294 (43.2)	224/486 (46.1)	351/780 (45.0)	124/284 (43.7)	228/504 (45.2)	352/788 (44.7)
*P *value	0.933^a^	0.787^a^	0.919			
rs1799836						
A	225/294 (76.5)	366/486 (75.3)	591/780 (75.8)	242/284 (85.2)	425/504 (84.3)	667/788 (84.6)
G	69/294 (23.5)	120/486 (24.7)	189/780 (24.2)	42/284 (14.8)	79/504 (15.7)	121/788 (15.4)
*P *value	0.008^a^	0.0004^a^	0.00001			

^a^*P *values are corrected for multiple testing, differences reaching statistical significance are *P *< 0.025.

Results of single-locus analysis are shown in Table 2. No significant differences are observed in frequency distributions of rs6323 between total (male and female) case and control groups (*P *= 0.919, Power = 100% when α = 0.05), or between each gender case and control groups (male: *P *= 0.933, Power = 99.9% when α' = 0.025; female: *P *= 0.787, Power = 99.9% when α' = 0.025). In contrast, allele frequency for the rs1799836 polymorphism displayed significant statistical difference between total case and control groups (*P *= 0.00001), identifying the G allele as a risk factor for schizophrenia (OR = 1.578, 95%CI = 1.284 - 1.938). Stratification of the analysis by gender revealed that the differences were significant in both female (*P *= 0.0004; OR = 1.764, 95%CI = 1.285 - 2.421) and male (*P *= 0.008; OR = 1.586, 95%CI = 1.121 - 2.245) groups. Odds ratios of female genotypes containing the G allele are listed in Table 3.

**Table 3:** Genotype distribution of rs1799836 in females


**Genotype**	**Schizophrenia (n, %)**	**Control (n, %)**	***P *value^a^**	**Odds Ratios (%95 Confidence Interval)**

AA	142/243 (58.4)	180/252 (71.4)	0.002	0.818 (0.717 - 0.933)
AG	82/243 (33.7)	65/252 (25.8)	0.061	1.308 (0.995 - 1.720)
GG	19/243 (7.8)	7/252 (2.8)	0.014	2.814 (1.204 - 6.575)

^a^Power for genotype distribution test is 99.9% when α = 0.05.

### LD and risk haplotype analysis

The *MAOA *and *MAOB *genes are located within 36.9 kb of each other on the X chromosome. As risk haplotype analysis displays an important role for genetic associations in the etiology of complex diseases [29], we performed an analysis of rs6323 (G/T) and rs1799836 (A/G). Using female data, the LD coefficient values of rs6323 and rs1799836 were tested in patients (D' = 0.387) and healthy controls (D' = 0.198) respectively. Bonferroni correction identified the rs6323T - rs1799836G haplotype as a risk factor for developing schizophrenia in female patients (Table 4). No association was observed in male groups (*P *= 0.235, Power = 99.9% when α = 0.05; OR = 1.359, 95% CI = 0.853 - 2.165).

**Table 4:** Haplotype distribution in female patients and controls


**Haplotype**	**Schizophrenia (n, %)**	**Control (n, %)**	***P *value^a^**	**Odds Ratio (95% Confidence Interval)**

G-A	222.36/486 (45.8)	241.28/504 (47.9)	0.504	0.918 (0.715~1.179)
G-G	39.64/486 (8.2)	34.72/504 (6.9)	0.449	1.200 (0.747~1.928)
T-A	143.64/486 (29.6)	183.72/504 (36.5)	0.021	0.731 (0.560~0.955)
T-G	80.36/486 (16.5)	44.28/504 (8.8)	0.0002	2.057 (1.392~3.039)

^a^*P *values are corrected for multiple testing, differences reaching statistical significance are *P *< 0.0125.

## Discussion

Complex disease may be caused by high-order risk interactions between or within genes. Here, we investigated the association of rs6323 and rs1799836 polymorphisms with schizophrenia in a Han Chinese population. Our data indicates that rs1799836 is a risk allele in the total population, and rs6323T - rs1799836G is a risk haplotype in females. As all genetic samples were obtained from geography-matched Han Chinese, these data are more definitive than results obtained by meta-analysis. The possibility of population stratification, however, raises some possibility for the identification of false positive associations. Future studies that incorporate a genomic control for sample selection would further increase the reliability of results. As such, we recommend that prior to global analysis of the roles of rs6323 and rs1799836 polymorphisms in schizophrenia, genetic associations should be performed strictly within each of the major populations including European, African, and East Asian analyses.

The *MAOA *gene, also called the "warrior" or "violence" gene [30], was identified as a candidate susceptibility gene for schizophrenia and aggressive behavior [31], although the risk polymorphism has not been confirmed [32]. Similarly, the T allele of the functional rs6323 locus was reported to be a risk factor in susceptibility to schizophrenia in Chinese men [16]. In our data, the T allele distributed almost equally among case and control samples, suggesting that rs6323 is not associated with schizophrenia. As such, our data contrast with a previous report by Qiu et al., however we attribute this discrepancy to the difference in sample size between the two studies. Based upon our findings, we suggest that focus be shifted to other polymorphisms, including those that distribute to coding regions and others that distribute to regulatory regions, both of which can affect functional gene expression.

The rs72554632 mutation, in which glutamine is exchanged with a termination codon, has been indentified in male patients with Brunner syndrome [33]. This mutation significantly diminishes MAOA activity, and is believed to contribute to the development of aggressive behavior characteristic of this syndrome. Previously, no association has been reported for rs72554632 in the development of schizophrenia. The proximity of rs72554632 4 bp upstream of rs6323, however, warrants mention. As a side outcome of genotyping rs6323 in this study, we investigated the prevalence of rs72554632 mutations in our cohort. We were unable to identify any mutations at the rs72554632 site, suggesting that no association exists with schizophrenia.

Although the A/G substitution of intron 13 (rs1799836) of *MAOB *does not change the amino acid sequence, the G allele has been associated with lower enzyme activity in human brain [23]. In line with the hypothesis of abnormal prefrontal dopamine signaling in schizophrenia, the association of rs1799836G allele with schizophrenia has been tested in a Spanish population [9,34]. Further, the G allele of rs1799836 was identified as a risk factor for developing schizophrenia in women. Our data confirms these results in a Han Chinese population. The observed differences in G allele frequency are statistically significant in both male and female patients compared to controls. The risk allele could be in LD with rs1799836. These data implicate *MAOB *in the etiology of schizophrenia, and suggest that additional polymorphisms of *MAOB *should be included in future studies [35].

Some variants that have no significant differences at the single-locus levels may be associated with disease upon haplotype analysis [36]. Weak linkage disequilibrium was found in controls between rs6323 and rs1799836, indicating frequent recombination in the Chinese population. A risk rs6323T - rs1799836G haplotype was found in female groups, although rs6323 alone was not identified as a risk factor in our study. Growing evidence indicates that gender-specific effects of *MAOA *and *MAOB *exist in various psychiatric diseases [9,12,24], and haplotype analyses support this gender-specific hypothesis [12]. Inactivation of the × chromosome in women during embryogenesis, however, makes it difficult to predict an allele's contribution to the risk for developing disease. As such, future studies should account for potential gender differences in the risk of schizophrenia.

## Conclusions

Although both *MAOA *and *MAOB *genes have been suggested as genetic factors in the pathogenesis of schizophrenia, our findings only support the association of *MAOB *polymorphism with susceptibility to schizophrenia in Han Chinese. Further, we identified rs6323T - rs1799836G as a risk haplotype in females. Future studies should be performed to validate these results, utilizing greater sample populations and incorporating additional polymorphisms of the *MAOA *and *MAOB *genes.

## Competing interests

The authors declare that they have no competing interests.

## Authors' contributions

YLW performed the genotyping, analyzed the data and drafted the manuscript, SBL offered the subjects and information, CXL analyzed the data and modified the manuscript, LH conducted experiments, and the corresponding author YL designed the study and finalized the manuscript. All authors have read and given final approval of the final manuscript.
